# Supplementary material for: Structural Insight into Integrin Recognition and Anticancer Activity of Echistatin
Source: Toxins (Basel). 2020 Nov 9;12(11):709. doi: 10.3390/toxins12110709 (PMC7695343; doi:10.3390/toxins12110709)
Supplement: Supplementary file 1 [file toxins-12-00709-s001.pdf]

Supplementary Materials: Structural Insight into Integrin Recognition and Anticancer Activity of Echistatin

Yi-Chun Chen, Yao-Tsung Chang, Chiu-Yueh Chen, Jia-Hau Shiu, Chun-Ho Cheng, Chun-Hao Huang, Ju-Fei Chen and Woei-Jer Chuang

Table S1. Summary of the interactions between Ech (chain A and chain B) and sulfate ion.

SO_4^{2-}	Ech's chain A	Ech's chain B	Interaction Atoms	Distance (Å)
O2	S4		CA	3.8
O2	S4		N	2.8
O2	S4		OG	3.4
O4	S4		OG	2.7
		K21	NZ	3.4
O2		K21	NZ	3.1
O3		R41	NE	2.6
O4		R41	NH2	3.0
O1		K45	NZ	2.9
O3		K45	NZ	3.6

Table S2. Summary of the interactions in the RGD loop, C-terminus, and between the RGD loop and C-terminus in Ech chain A.

Ech Residues	RGD Loop Residues	C-Terminus Residues	Interaction Atoms	Distance (Å)
K21	D30		O : N	2.9
K21	D30		N : O	3.2
A23	D29		N : OD1	2.9
R24	D27		O : N	3.2
D30		N42	OD1 : N	3.0
D30		K45	OD1 : NZ	3.0
N42		P43	OD1 : N	3.3
N42		H44	OD1 : N	2.9

Table S3. Summary of the interactions in the RGD loop, C-terminus, and between the RGD loop and C-terminus in Ech chain B.

Ech Residues	RGD Loop Residues	C-Terminus Residues	Interaction Atoms	Distance (Å)
K21	D30		O : N	2.8
K21	D30		N : O	3.1
A23	D29		N : OD1	2.7
R24	D26		NE : OD1	2.9
R24	D26		NE2 : OD2	2.9
R24	D27		O : N	2.9
M28		K45	O : NZ	2.7
D30		N42	OD1 : N	2.7
D30		K45	OD1 : NZ	2.8
N42		P43	OD1 : N	3.2
N42		H44	OD1 : N	2.8

Table S4. Summary of the interactions in the RGD loop, C-terminus, and between the RGD loop and C-terminus in trimestatin.

Trimestatin Residues	RGD Loop Residues	C-Terminus Residues	Interaction Atoms	Distance (Å)
R46	D55		O : N	2.9
A48	D54		N : OD1	3.0
R49	F52		O : N	4.5
D55		W67	OD1 : N	3.0
W67		N68	NE1 : O	3.5

Table S5. Summary of the interactions between Ech chain A and αvβ3 integrin.

Ech	αvβ3		Interaction	Distance (Å)
Chain A	αv	β3	Atoms	
R22		N313	HH22 : O	1.82
R24	D150		NE : OD1	2.79
R24	D150		NH2 : OD1	3.16
R24	D150		HE : OD1	2.49
R24	D218		HH11 : OD2	1.78
R24	D218		NH1 : OD1	2.71
R24	D218		NH1 : OD2	2.73
R24	D218		NH2 : OD2	3.14
R24	D218		HH21 : OD2	2.37
G25	D218		N : OD1	3.53
D26		S121	OD1 : OG	3.11
D26		Y122	OD2 : N	2.83
D26		S123	O : N	3.55
D26		S123	OD1 : N	3.04
D26		S123	OD1 : OG	2.68
D26		N215	OD2 : N	2.91
D26		N215	OD2 : HD21	2.47
D26		R216	N : O	3.21
D26		D217	N : O	3.06
Y31		D126	HH : OD2	1.68
H44		Y122	O : HH	2.06

Table S6. Summary of the interactions between Ech chain B and αvβ3 integrin.

Ech	αvβ3		Interaction	Distance (Å)
Chain B	αv	β3	Atoms	
R24	D150		NE : OD1	2.64
R24	D150		NH2 : OD1	2.59
R24	D150		HE : OD1	1.99
R24	D150		HH22 : OD1	1.95
R24	D218		NH1 : OD1	2.63
R24	D218		NH1 : OD2	2.63
R24	D218		NH2 : OD2	3.67
R24	D218		HH12 : OD2	1.85
D26		S121	OD1 : OG	2.68
D26		Y122	OD2 : N	2.77
D26		S123	O : N	3.08
D26		S123	OD1 : N	2.88
D26		S123	OD1 : OG	2.73

D26	N215	OD2 : N	3.01
D26	R216	N : O	3.60
D26	D217	N : O	3.08
M28	D126	SD : N	3.72
Y31	D126	HH : OD2	2.13
K45	M180	HZ2 : SD	2.30

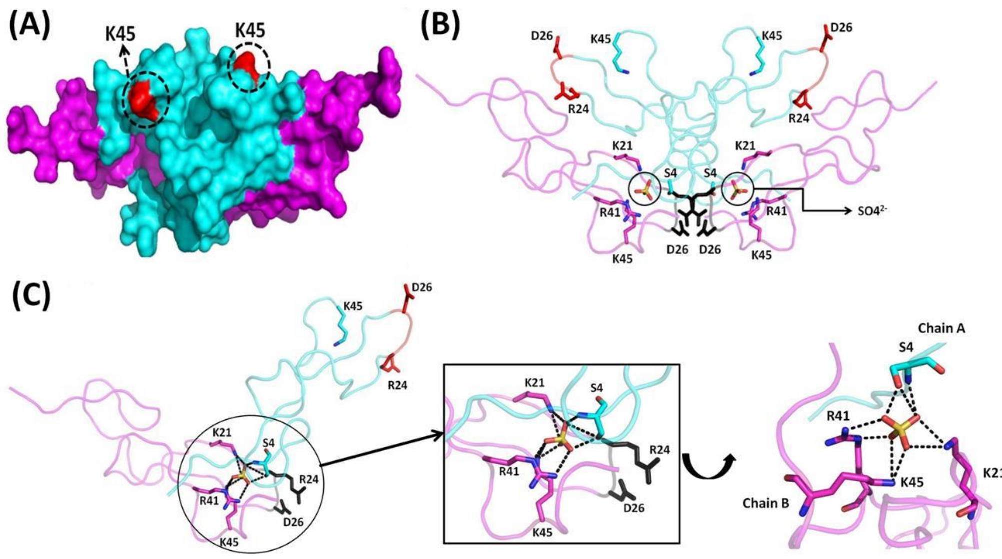

Figure S1. Crystal packing of Ech. (**A**) The crystal structure of Ech is shown as a surface in PyMOL, and Ech chain A and chain B are shown in cyan and magenta, respectively. K45 in chain A is colored red; (**B**) The RGD loop of chain B is squeezed by the contacts from crystal packing. R24 and D26 in chain A are colored in red. R24 and D26 in chain B are colored in black. The sulfate ion is colored yellow; (**C**) Asymmetric chain A and chain B can interact with the same sulfate ion. Residue S4 of chain A and residues K21, R41 and K45 of chain B make interactions with sulfate ions.

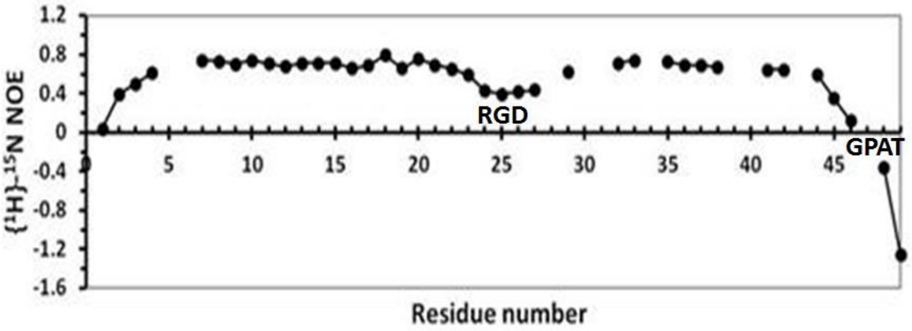

Figure S2. ^{1}H-^{15}N heteronuclear NOE values measured for individual amide sites along the backbones. The gaps are from the proline residues. Residues R24-D27 in the RGD loop and K45-T49 in the C-terminus had NOE values less than the average NOE value of Ech.

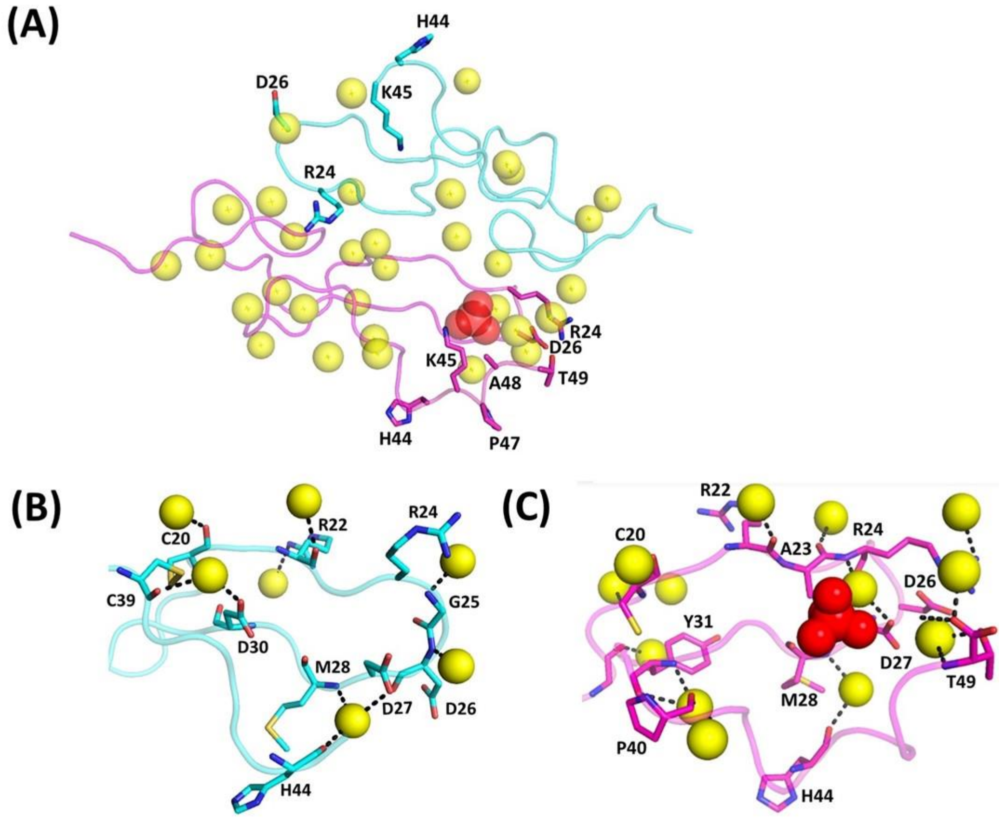

Figure S3. The interactions of Ech with water molecules and sulfate. (**A**) Overall schematic diagram of Ech crystal structure interacting with water molecules and sulfate. Water molecules are colored yellow, sulfate is colored red, Ech chain A is colored cyan, and Ech chain B is colored magenta; (**B**) RGD loop residues D27 and M28 of Ech chain A connected to the C-terminal residue H44 through the water molecules. Moreover, the side chain of residue D30 also linked the main chain of residue D39 through the water molecule; (**C**) The water molecule connected the RGD loop residues R24 and D27 of Ech chain B, which also brought the RGD loop residue M28 to the C-terminal residue H44 close to each other, and the C-terminal residue T49 interacted with the water molecules to stabilize the C-terminal tail structure.
